# Urban–Rural Disparities in School Lunch Food Composition: Evidence from 12.7 Million Dish Records in Taiwan’s National School Food Registry

**DOI:** 10.3390/nu18172871

**Published:** 2026-09-02

**Authors:** Hui-Min Wang, Jiun-Hao Wang, Kuo-Chen Wu

**Affiliations:** 1 Department of Bio-Industry Communication and Development, National Taiwan University, No. 1, Sec. 4, Roosevelt Rd., Taipei 106, Taiwan; d03630004@ntu.edu.tw; 2 Graduate Institute of Mass Communication, National Taiwan Normal University, 7F, General Building, No. 129-1, Sec. 1, Heping E. Rd., Taipei 106, Taiwan; robinsixrainbow@gmail.com

**Keywords:** school meals, child nutrition, urban–rural disparities, dietary patterns, ultra-processed food, whole-grains, public food procurement, Taiwan

## Abstract

***Background***/***Objectives:*** For many schoolchildren—particularly those in low-resource areas—the school lunch is the most reliable balanced meal of the day, so its composition is a direct determinant of dietary equity. The aim of this study was to quantify urban–rural disparities in school lunch food composition at national scale and to test whether they persist once county socioeconomic structure is controlled. ***Methods:*** Using 12,688,008 dish records from 3894 schools across all 22 Taiwanese counties over 24 months (May 2024–April 2026), retrieved from the Ministry of Education’s School Food Registry Platform, we constructed four dish-level dietary composition indicators—plant-forward, organic, processed, and whole-grain shares—and analyzed county-month panels (N = 418) using hierarchical regression with weighted least squares and heteroskedasticity-consistent standard errors. ***Results:*** The four dimensions followed distinct urban–rural patterns. Plant-forward provision exhibited a stable urban-to-rural decline. Organic and whole-grain provision displayed a suppressor structure: rural disadvantage disappeared—and reversed—once county income was controlled, revealing latent local supply capacity masked by income disparities. Processed-dish use, once income and population density were accounted for, was concentrated in lower-income, more densely populated counties rather than rural ones, indicating an urban concentration pattern rather than a rural deficit. A 2017 traceable-food pilot policy still exhibited a legacy effect on organic procurement seven years after its nationwide rollout. ***Conclusions:*** School meal programs thus deliver systematically different dietary environments to children in different places. Narrowing these gaps, rather than raising national averages, should be the core criterion for evaluating school lunch reform, with interventions differentiated across dietary dimensions.

## 1. Introduction

Urban–rural disparities in child health remain a persistent policy concern in Taiwan, as in many high-income countries. Childhood overweight and obesity exhibit marked but shifting geographic patterns: earlier national surveys found a higher prevalence among urban children, whereas more recent metropolitan studies reported an elevated risk among peri-urban children of lower parental socioeconomic status [1,2]. This evidence suggests that the geography of child health in Taiwan is structured by socioeconomic composition rather than by location alone. These gaps are not reducible to individual preferences; they reflect cumulative structural differences in food supply chains, household resources, school facilities, and local industrial composition. Within this landscape, school lunches occupy a unique position. For children in low-resource areas, the school lunch is often the single most reliable balanced meal of the day, which makes it one of the few policy levers through which a government can act directly on the dietary intake of every child, regardless of household resources.

A substantial international evidence base positions schools as central settings for promoting healthy eating and preventing childhood obesity [3], and the ‘school food revolution’ literature documents how reform of public school meal provision can improve the quality of children’s diets while serving broader sustainability goals [4]. Because attendance is near-universal and the meal is consumed daily, school lunch composition defines a dietary environment encountered by every enrolled child, independent of household purchasing power. Yet, most of what is known about that environment comes from surveys, menu audits of limited samples, or single-district case studies; population-scale evidence on how school meal composition actually varies across space remains rare.

The four dietary dimensions examined in this study are each relevant to child nutrition. Whole-grain intake is consistently associated with lower risks of cardiovascular disease and all-cause mortality in prospective studies [5], whereas high consumption of ultra-processed foods—industrially formulated products such as reconstituted meat items—is linked to poorer diet quality and adverse health outcomes [6]. Economic constraints are known to push diets toward energy-dense, nutrient-poor options [7], which is precisely why publicly provided meals matter most for children whose households face them. Plant-forward provision speaks to both dietary quality and the environmental sustainability of school food systems, while organic provision—although not a nutrient claim—indexes the quality orientation of school procurement and its integration with certified supply chains. Whether these dimensions vary systematically between urban and rural schools is therefore a question of dietary equity, not of procurement logistics alone. Using an administrative census of Taiwan’s school lunch program, we ask how each dimension is distributed across the urban–rural spectrum and which structural factors—income, agricultural employment, population density, and prior policy exposure—account for the observed gaps. The aim of this study was to quantify urban–rural disparities in the food composition of Taiwan’s school lunches at national scale, and to test whether these disparities persist once county socioeconomic structure is controlled.

## 2. Materials and Methods

### 2.1. Study Setting and Policy Context

This is a nationwide cross-sectional ecological study of administrative census data, analyzed at the county-month level.

Taiwan comprises 22 county-level administrative units, ranging from dense metropolitan municipalities (e.g., Taipei City) to remote agricultural counties and outlying islands (e.g., Kinmen and Lienchiang). Following the Ministry of Education’s regulations on the designation of schools in remote areas, we classified these into three tiers: metropolitan (seven counties), suburban (six counties), and rural (nine counties). The full classification, together with a reference map, is provided in Supplementary Materials Figure S1 and Table S2, respectively.

Two institutional features make the Taiwanese case analytically feasible. The first is the School Food Registry Platform (校園食材登錄平臺), established in 2014 [8]. Central or on-site school kitchens must upload each day’s dishes and ingredient sources after lunch service; schools procuring certified domestic traceable ingredients receive a central incentive—introduced at NT$3.5 per student-meal in 2017 and raised from May 2022 to NT$10 per student per day (NT$14 for officially designated remote schools) [9,10]—and complete daily logging on the platform is the administrative condition for claiming it; the platform additionally performs automated consistency checks against supplier records. Compliance is therefore near-universal, and the resulting data constitute an administrative census of school lunches, rather than a sample. The Ministry of Education reports that certified traceable ingredients now account for 98.05% of school lunch ingredient items [11].

The second feature is the ‘3 Labels & 1 QR’ program (三章一Q), a traceable-food procurement policy referring to three national agri-food certification labels—Traceable Agricultural Products (TAPs), CAS premium agricultural products, and certified organic—plus a production-traceability QR code. In February 2017, the Council of Agriculture launched the program as a pilot in six counties (Taichung City, Tainan City, Hsinchu County, Taitung County, Yilan County, and Hsinchu City), extending it nationwide in September 2018 [12]. The seven-year interval between the pilot and our observation window allows us to test whether early adoption left a durable institutional imprint—a question that speaks directly to the path-dependence literature in policy studies [13].

The incentive is distinct from the financing of school lunches themselves, which is decentralized: meal fees are borne primarily by parents and local governments, per-meal prices vary substantially across counties, and several county governments operate fully subsidized (“free lunch”) programs, while central support targets disadvantaged students and remote-area kitchen infrastructure [9,11]. This decentralized financing is one reason the county was chosen as the unit of analysis.

The four dimensions studied here correspond to explicit anchors in Taiwan’s regulatory framework rather than ad hoc researcher choices. Article 23 of the School Health Act requires school meals to follow the Ministry of Education’s School Lunch Food Content and Nutritional Standards, mandates that a vegetarian option be offered, and requires priority procurement of certified domestic agricultural products [14]; the ‘3 Labels & 1 QR’ guideline is issued under this article together with Article 11 of the Food and Agriculture Education Act [15]. The Nutritional Standards (revised 28 December 2020) additionally advise minimizing fish- and meat-based semi-processed products, cap deep-fried items at twice per week and sugary desserts at once per week, and specify daily food-group composition including whole and unrefined staples [16]. Plant-forward, organic/traceable, processed, and whole-grain shares therefore index compliance-relevant dimensions of the national policy framework.

### 2.2. Data

We retrieved the ‘lunch dish’ dataset from the Registry Platform’s open API (v1) in April 2026, downloading 1056 files spanning 22 counties × 2 school levels × 24 months (May 2024–April 2026), totaling 12,966,481 raw records. After normalizing school names into stable identifiers and removing duplicates (same school, date, and dish name), 12,688,008 valid records from 3894 schools remained, organized as 80,990 school-month cells. School-month cells with fewer than five serving days in a given month were excluded, reducing the 80,990 cells to 73,536. These were then aggregated to the county-by-month level—pooling both school levels (elementary/junior-high and senior-high)—yielding 418 county-month observations for the main analysis; county-months in which no school met the five-serving-day threshold (most often small rural counties in recess-adjacent months) dropped out at this step. July, August, and February were treated as recess months (summer and Lunar New Year).

Both school levels operate under the same statutory framework (the School Health Act) and within the same county-level procurement environment, and the registry covers them identically; levels were therefore pooled when aggregating to county-months, treating the county school food environment as the unit of interest. As a robustness check, all models were re-estimated on elementary and junior-high schools only—the compulsory-education tier, for which lunch provision is near-universal—with substantively unchanged results (Supplementary Table S3).

### 2.3. Outcome Variables

Four dish-level binary indicators were constructed using rule-based Chinese keyword matching and then aggregated to county-month shares. Plant-forward dishes comprise genuine soy-based items (tofu, dried tofu, soy milk) and plant-based meat analogs (e.g., vegetarian ‘chicken’ or ‘ham’); analogs are simultaneously coded as processed, reflecting their dual character. Organic dishes are identified by organic, TAP/traceability, and ‘3 Labels & 1 QR’ markers. Processed dishes are restricted to ready-made industrial items (nuggets, fish balls, sausages, ham, and bacon); freshly fried whole foods (e.g., fried chicken leg and fried sweet potato) are excluded so that the indicator captures the product form rather than the cooking method. Whole-grain dishes are identified by brown rice, multigrain, oats, quinoa, and similar markers. Items offered as ‘fresh milk or soy milk’ (a statutory either/or beverage provision) are not coded plant-forward. Classification validity was assessed against a manually adjudicated random sample of 200 dishes drawn with an independent seed; Cohen’s kappa for each dimension is reported in Section 3.1.

All four indicators are frequency-based: each record corresponds to one dish item listed on a school’s daily menu, and each indicator is computed as the share of dish records in a school-month that match the dimension’s dictionary. The registry does not record portion weights or served quantities; the indicators therefore capture how often a category appears on menus rather than the amount consumed. Table 1 presents worked classification examples for each dimension, including boundary cases resolved during manual adjudication.

### 2.4. Explanatory Variables

The focal variable is the three-tier urbanicity classification (urban as a reference; suburban and rural dummies). Controls were entered hierarchically in three blocks: (i) month fixed effects; (ii) county socioeconomic structure—mean household income from the 2024 Family Income and Expenditure Survey (published October 2025; covering 20 of 22 counties, with Kinmen and Lienchiang imputed from historical county data), agricultural employment share, and population density, all z-standardized; and (iii) a 2017 pilot-county indicator for the ‘3 Labels & 1 QR’ program.

### 2.5. Estimation

For each of the four outcomes, we estimated a four-step hierarchical regression [17,18] at the county-month level (N = 418), entering month effects, urbanicity, socioeconomic structure, and the pilot indicator sequentially, and tested each increment with an F-test on ΔR^2^. Because school counts differ widely across counties (as few as roughly six contributing schools per month in Lienchiang versus up to about 320 in Kaohsiung), models are estimated by weighted least squares with school counts as weights and HC3 heteroskedasticity-consistent standard errors. Intraclass correlations at the county level [19] were computed to characterize the clustering structure of the panel.

All analyses were performed in Python 3.13.9 (Python Software Foundation, Wilmington, DE, USA) using the pandas 2.3.3, NumPy 2.3.5, and statsmodels 0.14.5 packages.

## 3. Results

### 3.1. Classification Validity

Two raters independently coded an additional random sample of 200 dishes drawn with a fixed seed (2026) that was held out from and statistically independent of the seed-42 sample used during rule development. This separation ensured that validity was not assessed on the data that informed keyword rules. The agreement between the automated classifier and manual adjudication was high across all four dimensions. Cohen’s kappa was 1.000 for processed dishes, 0.931 for organic dishes, 0.911 for plant-forward dishes, and 0.855 for whole-grain dishes, with raw agreement of 0.985 or higher in every case. According to the Landis–Koch benchmarks, all four coefficients fall in the “almost perfect” range (>0.81) and well above the 0.61 threshold for substantial agreement, supporting the construct validity of the rule-based indicators. The few discrepancies were isolated and dimension-specific: for whole-grain dishes, the single false negative was a barley-rice item (barley is not in the whole-grain keyword list; see Section Limitations), and for plant-forward dishes, the two false negatives were branded soy milk products whose names did not contain a soy keyword.

### 3.2. Descriptive Urban–Rural Patterns

**Figure 1:**
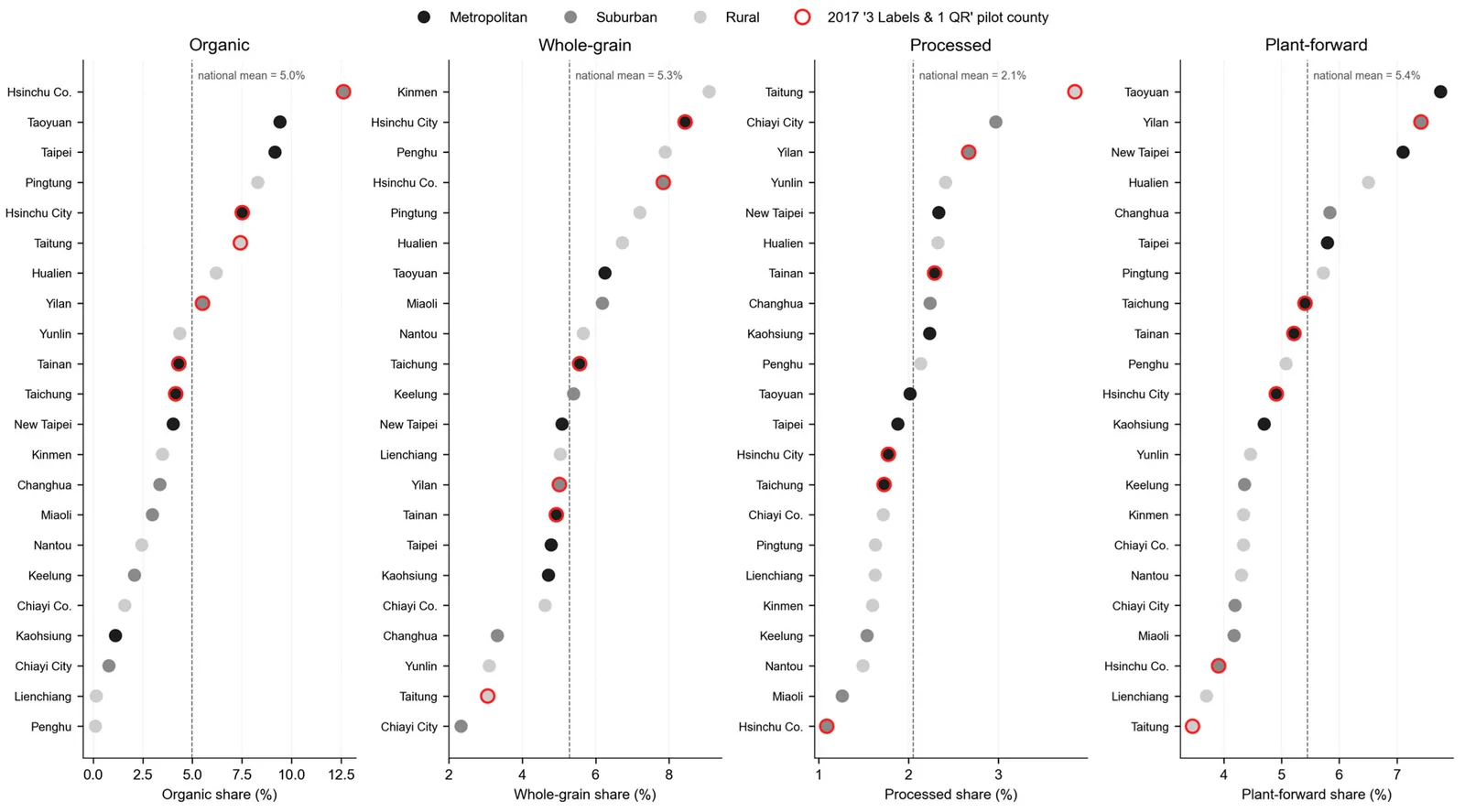
County-level ranking of the four food composition shares across Taiwan’s 22 counties (monthly means, May 2024–April 2026). Each panel ranks counties on one outcome; marker shade denotes urbanicity tier (metropolitan, suburban, rural) and red rings mark the six 2017 ‘3 Labels & 1 QR’ pilot counties. The dashed lines represent school-weighted national means.

Figure 1 ranks all 22 counties based on the four outcome shares. Across the analytic sample, school-weighted national means were 5.44% for plant-forward dishes, 5.05% for organic dishes, 5.35% for whole-grain dishes, and 2.01% for processed dishes; thus, processed dishes were the least common of the four categories. These four dimensions did not share a common spatial signature. Plant-forward use followed a monotonic urban-to-rural decline (metropolitan 5.90%, suburban 5.16%, and rural 4.80%). Organic use showed no simple gradient: the highest county shares were a heterogeneous mix spanning a suburban county (Hsinchu County, 12.6%), metropolitan municipalities (Taoyuan 9.4%, Taipei 9.2%), and rural counties (Pingtung 8.4%, Taitung 7.4%), while the lowest shares were the two outlying-island counties (Penghu 0.1%, Lienchiang 0.2%); this mix foreshadows the income-suppression structure recovered in the regressions. Whole-grain use was led conspicuously by outlying-island and rural counties (Kinmen 9.1%, Penghu 7.9%) alongside Hsinchu City. Processed use was highest in Taitung (3.9%) and lowest in the suburban counties of Hsinchu (1.1%) and Miaoli (1.2%). As shown in Section 3.3, this raw ranking inverts once county income and population density are held constant; therefore, the apparent rural concentration of processed dishes is not a locational effect.

### 3.3. Does the Urban–Rural Gap Survive Socioeconomic Controls?

Table 2 shows the suburban and rural coefficients across the four hierarchical steps for each outcome. Three distinct patterns have emerged. First, the plant-forward gap is a genuine locational gradient that persists, in attenuated form, under controls: relative to metropolitan counties, suburban and rural shares are lower at every step, and in the full model, the rural coefficient remains negative and significant (B = −0.649, *p* < 0.001; suburban B = −0.496, *p* < 0.001). Adding socioeconomic structure in Step 3 shrinks these gaps by roughly half rather than eliminating them, and county income enters with a significant negative coefficient (B = −0.325, *p* < 0.001). Pilot counties, moreover, showed significantly lower plant-forward shares (B = −0.412, *p* < 0.01), and the pilot step added significant explanatory power (ΔR^2^ = 0.031, F = 16.28, *p* < 0.001). Second, organic and whole-grain use displayed a suppressor structure. In the urbanicity-only model (Step 2), suburban and rural coefficients were non-significant and, for organic, slightly negative; once county income was entered in Step 3, the rural coefficients became large and positive (organic rural B = +4.56, whole-grain rural B = +2.87, both *p* < 0.001), and even the suburban organic coefficient turned positive (B = +1.55, *p* < 0.001). Income is the suppressor: it correlates positively with both outcomes and negatively with rurality, so holding it constant reveals a latent rural advantage that the raw shares conceal. The socioeconomic block dominated the explained variance for both outcomes (organic ΔR^2^ = 0.341; whole-grain ΔR^2^ = 0.483; both *p* < 0.001), and the whole-grain model was the best-fitting of the four (R^2^ = 0.501). Third, processed use is structured by socioeconomic composition rather than location. The rural coefficient is essentially zero in Step 2 (B = +0.059, n.s.), but becomes significantly negative once income and density are controlled (full-model rural B = −0.236, *p* < 0.05; suburban B = −0.303, *p* < 0.001). Processed shares are higher in lower-income and more densely populated counties (income B = −0.477, density B = +0.121, both *p* < 0.001).

### 3.4. Heterogeneity Across Dietary Dimensions

**Figure 2:**
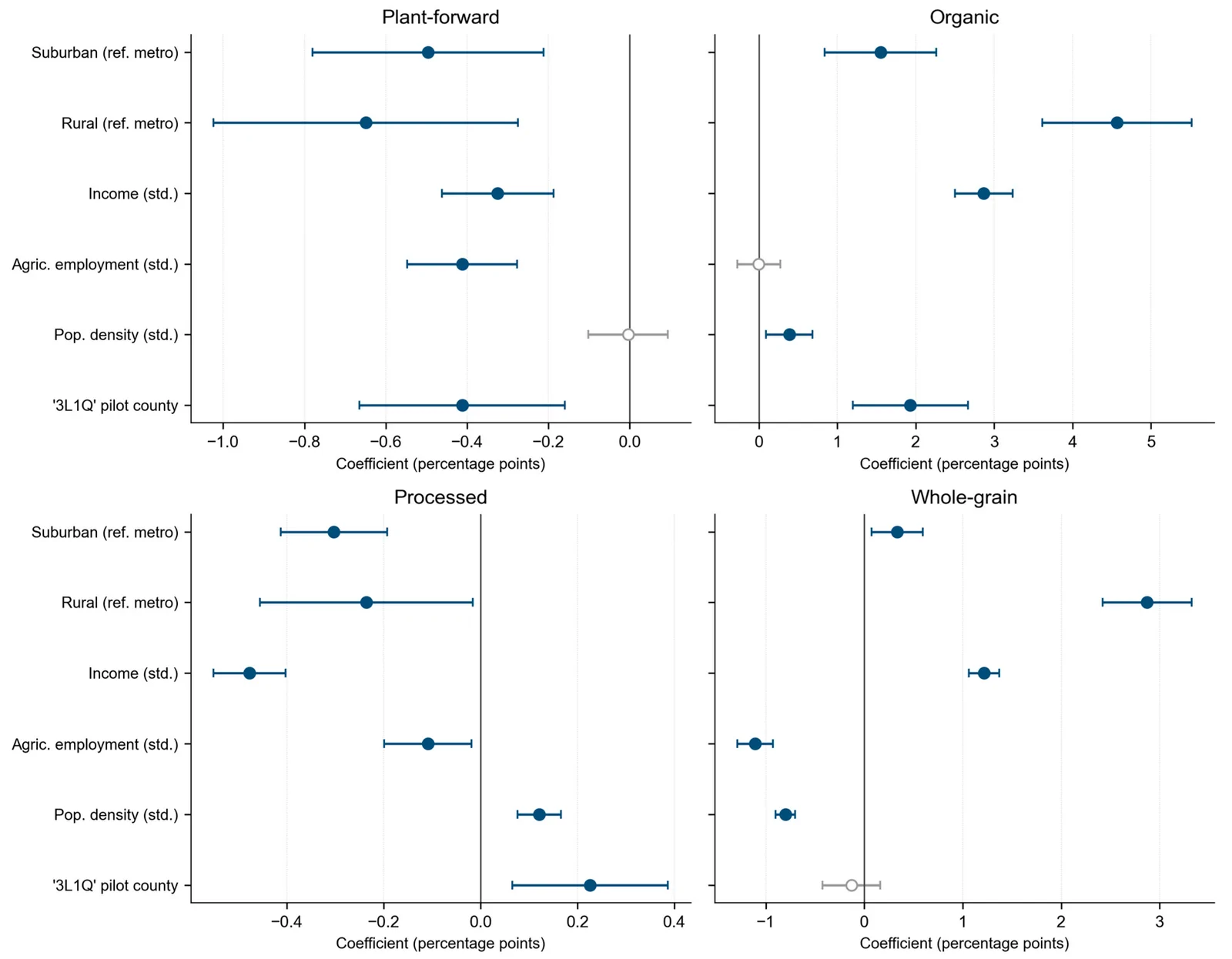
Final model (Step 4) regression coefficients with 95% confidence intervals for the four food composition outcomes (county-month panel, N = 418; WLS with school-count weights and HC3 standard errors). The horizontal axis is the coefficient in percentage points; the vertical line marks zero, and an interval crossing zero indicates a coefficient that is not significant at the 0.05 level.

**Figure 3:**
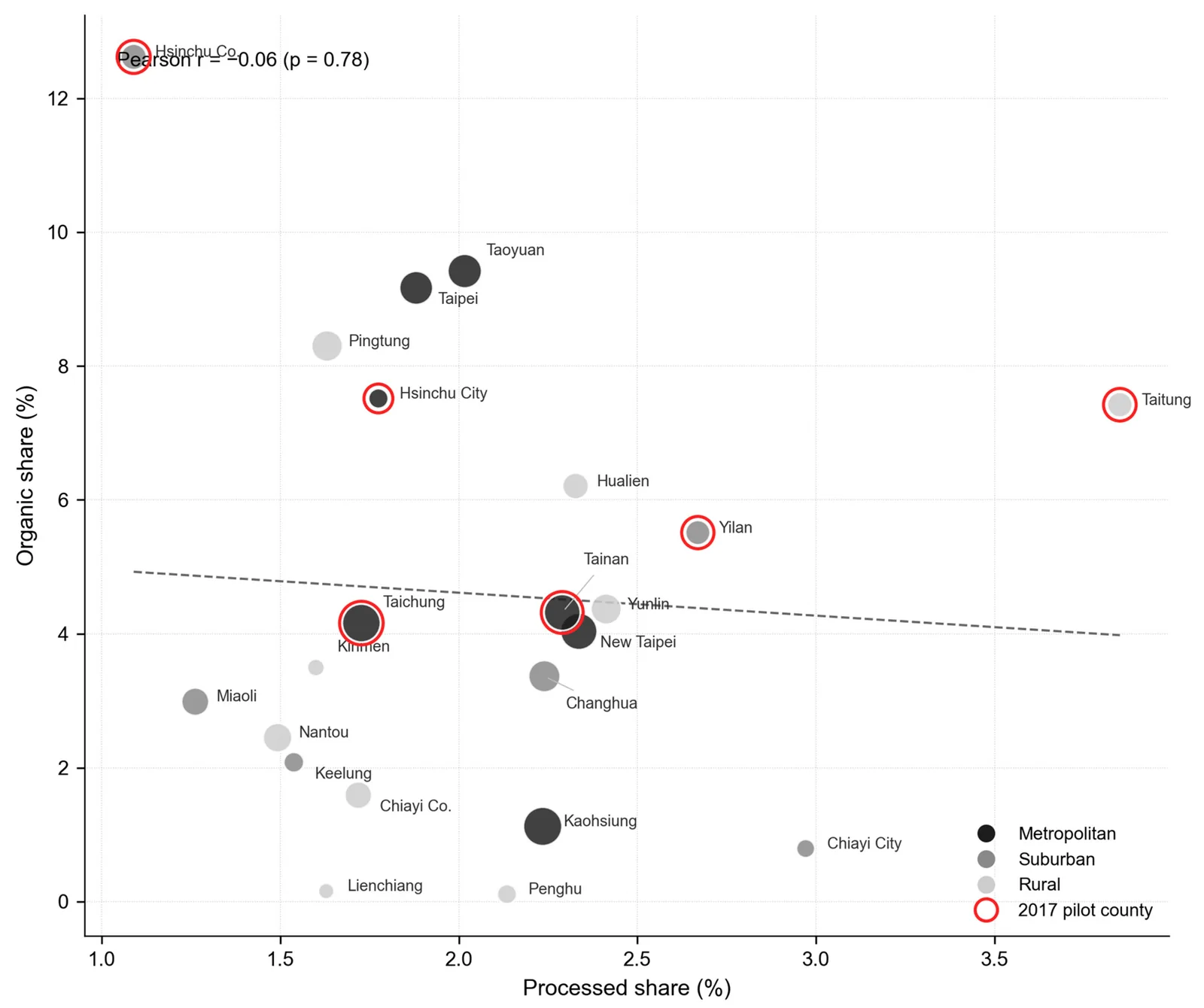
County-level joint distribution of organic and processed shares (N = 22 counties). The marker size is proportional to the county’s school count; the dashed line is the regression fit. The two shares were essentially uncorrelated (Pearson r = −0.01, *p* = 0.98), indicating that organic and processed procurement varied independently across counties.

Figure 2 and Figure 3 compare the final model coefficients and the county-level joint distribution of organic and processed shares. Collectively, the four final models show that the dietary dimensions respond to different structural drivers rather than moving as a single “healthy-eating” bundle. The rural coefficient alone reverses sign across outcomes—negative for plant-forward (−0.65) and processed (−0.24) but strongly positive for organic (+4.56) and whole-grain (+2.87)—so no single ranking of rural counties as “better” or “worse” is tenable. Income behaves similarly, suppressing organic and whole-grain shares when uncontrolled, yet pushing processed shares down once entered. The clearest illustration is the county-level joint distribution of organic and processed shares: across the 22 counties, these two shares are essentially uncorrelated (Pearson r = −0.01); therefore, a county’s position on processed-food use carries almost no information about its organic procurement. The four-quadrant pattern therefore contains counties of every type—high-organic/low-processed (e.g., Hsinchu County), low-organic/high-processed (e.g., Taitung), and mixed profiles—rather than a single diagonal trade-off. This orthogonality reinforces the central message of Section 3.3: urban–rural disparities in school lunch composition are dimension-specific, and interventions calibrated to one dimension cannot be assumed to move the others. One coefficient warrants caution: the population density term for organic procurement, non-significant in Step 3, becomes positive and significant in the full model (B = +0.387, *p* < 0.05); given this sensitivity to specification, the organic–density association is not interpreted substantively here (see Section Limitations).

## 4. Discussion

Three findings warrant emphasis. First, the urban–rural gap in school lunch composition is real but dimension-specific: a single ‘rural deficit’ narrative fits the plant-forward indicator yet misdescribes the other three dimensions. Second, for organic and whole-grain procurement, the apparent rural disadvantage is an artifact of income: once county income is held constant, rural counties lead—consistent with the view that remote areas possess latent agricultural capacity (smallholder organic production, island grain cultures) suppressed by fiscal constraints rather than by absent supply, echoing the established link between household income and dietary quality [7]. This reading aligns with the neighborhood-effects literature [20] and with the public food procurement scholarship that treats school meals as an instrument of sustainable development [4,21,22].

These patterns speak to an international policy conversation. Brazil’s national school feeding program (PNAE) reserves at least 30% of federal school food funds for procurement from family farming under Law 11,947 of 2009, explicitly deploying school meals as an instrument of rural development and food security [23]. Organic procurement initiatives in Danish schools [22] and the municipal school meal reform of Rome [21], like farm-to-school programs in the United States [24,25], likewise treat school food as a lever connecting child nutrition to local agriculture. Taiwan’s configuration—universal registry-based transparency combined with a traceability incentive—adds a distinctive case to this landscape: it shows that even under a uniform national framework, decentralized financing and county socioeconomic structure generate dimension-specific urban–rural gaps, and that rural capacity can remain latent unless procurement support offsets income constraints.

The following policy implications emerge from this study. School lunch evaluation should adopt gap-narrowing—not national averages—as its primary criterion; rural organic and whole-grain potential should be unlocked through targeted procurement subsidies that offset income constraints; and processed-food reduction should be aimed at the ‘urbanized but less affluent’ county profile through supply-side intervention with catering contractors, rather than at rural areas per se [3,24,25].

By “reframing” we mean three specific contexts. First, evaluation metrics: progress should be assessed by the narrowing of urban–rural gaps rather than by national averages, which the suppressor structure shows can mask rural capacity. Second, procurement subsidies: support should be differentiated by county fiscal capacity, since identical per capita incentives leave income-constrained counties unable to convert latent supply into menus. Third, supplier-side governance: because menus are substantially shaped by caterers and suppliers operating under cost pressure, reductions in processed items are more plausibly achieved through contract standards and supplier-facing incentives than through school-level exhortation.

### Limitations

Keyword classification, although validated by manual adjudication (Cohen’s kappa 0.855–1.000 across the four dimensions), measures dish naming rather than nutrient content. The four indicators are therefore best read as population-level dietary-pattern proxies—descriptors of the food environment that school meals place before children—rather than as estimates of individual nutrient intake; this is the level at which procurement decisions operate, and the level at which administrative census data can measure the food environment consistently and without self-report error. The keyword lists are also not exhaustive: validation identified a small number of items that fall outside the current dictionaries—most notably barley-rice dishes, which are not captured by the whole-grain keywords and produced a single false negative for that dimension—so the whole-grain share, in particular, is a marginally conservative lower bound; because such items are rare, the effect on county-month shares is negligible and does not alter the reported patterns. The population density coefficient for organic procurement is sensitive to specification and is not interpreted substantively. In addition, income for two outlying-island counties is imputed, and the design is cross-sectional, so coefficients describe associations. The pilot effect, in particular, cannot be separated from selection into the pilot; a difference-in-differences design exploiting the 2017 timing with pre-pilot data is the natural next step. Linking food composition to nutrient intake and ultimately to child health outcomes would complete the causal chain from the procurement structure to health equity.

Regulator-relevant extensions of the present dictionaries are nonetheless feasible within the same registry: the frequency of deep-fried items and of sugary desserts (which the National Standards cap at twice and once per week, respectively) [16], vegetable variety, linkage of dish records to the national food composition database, and a mapping of the processed dictionary onto the NOVA classification [6]. We regard these as the natural next step of this research program.

## 5. Conclusions

Using an administrative census of 12.7 million school lunch dishes, this study shows that Taiwan’s urban–rural gap in school food composition is substantial, dimension-specific, and—in the cases of organic and whole-grain procurement—a reversal in disguise: rural counties trail only because income suppresses the capacity they demonstrably possess. Reframing school lunch policy to close these gaps, with instruments differentiated by dietary dimension, would convert a daily public meal into a working tool for rural revitalization and child health equity. Concretely, policy design should specify minimum traceable-ingredient thresholds with registry-based verification; enforcement of the existing semi-processed and fried-frequency caps; minimum local and organic procurement shares calibrated to county agricultural capacity; subsidies differentiated by county fiscal capacity rather than uniform per capita rates; and supplier contract standards that make compliance commercially rational for caterers.

## Figures and Tables

**Table 1:** Worked classification examples for the four dish-level dimensions, including boundary cases resolved during manual adjudication.

Dish (Chinese)	English Gloss	Classification	Rationale
麻婆豆腐	Mapo tofu	plant-forward	soy product (tofu)
素雞	vegetarian “chicken”	plant-forward + processed	plant-based meat analog: dual-coded
貢丸湯	pork-ball soup	processed	ready-made industrial item
炸雞腿	fried chicken leg	(none)	freshly fried whole food: cooking method, not product form
糙米飯	brown rice	whole-grain	unrefined staple
香Q米飯	“chewy” white rice	(none)	polished rice
鮮奶或豆漿	milk-or-soy milk option	(none)	statutory either/or beverage provision, excluded from plant-forward
有機青江菜	organic bok choy	organic	certified-organic marker

**Table 2:** Full-model (Step 4) hierarchical regression coefficients for the four food composition outcomes (county-month panel, N = 418; WLS with school-count weights and HC3 standard errors). The entries are unstandardized coefficients in percentage points, with standard errors in parentheses. The reference category for urbanicity was metropolitan counties. Income, agricultural employment, and population density were standardized. The complete four-step estimates are presented in Supplementary Table S1.

Predictor	Plant-Forward	Organic	Processed	Whole-Grain
Suburban (ref. metropolitan)	−0.496 *** (0.145)	+1.552 *** (0.363)	−0.303 *** (0.056)	+0.334 * (0.133)
Rural (ref. metropolitan)	−0.649 *** (0.191)	+4.564 *** (0.486)	−0.236 * (0.112)	+2.872 *** (0.231)
Disposable income (std.)	−0.325 *** (0.070)	+2.868 *** (0.189)	−0.477 *** (0.038)	+1.216 *** (0.079)
Agricultural employment (std.)	−0.412 *** (0.069)	−0.001 (0.140)	−0.109 * (0.046)	−1.110 *** (0.092)
Population density (std.)	−0.004 (0.050)	+0.387 * (0.151)	+0.121 *** (0.023)	−0.801 *** (0.051)
‘3 Labels & 1 QR’ pilot county	−0.412 ** (0.129)	+1.931 *** (0.374)	+0.226 ** (0.082)	−0.130 (0.150)
Model R^2^ (full model)	0.261	0.411	0.261	0.501

Note: N = 418 county-months. * *p* < 0.05, ** *p* < 0.01, *** *p* < 0.001. The coefficients are in percentage points of the respective dish shares. Step-wise ΔR^2^ and incremental F-tests are reported in the text and in detail in Table S1.

## Data Availability

The raw dish records analyzed in this study are publicly available from the Ministry of Education’s School Food Registry Platform open API at https://fatraceschool.k12ea.gov.tw/ (accessed on 30 April 2026). The derived county-month analytic dataset and the analysis code are available from the corresponding author upon reasonable request.

## Supplementary Materials

The following supporting information can be downloaded at https://www.mdpi.com/article/10.3390/nu18172871/s1, Figure S1: Map and three-tier classification of Taiwan’s 22 counties; Text S1: Institutional description of the School Food Registry Platform; Text S2: Timeline of the ‘3 Labels & 1 QR’ program; Table S1: Full hierarchical regression tables for all four outcomes; Table S2: County classification table; Table S3: Hierarchical regressions re-estimated on elementary and junior-high schools only.
