# Supplementary material for: Urban–Rural Disparities in School Lunch Food Composition: Evidence from 12.7 Million Dish Records in Taiwan’s National School Food Registry
Source: Nutrients. 2026 Sep 2;18(17):2871. doi: 10.3390/nu18172871 (PMC13567730; doi:10.3390/nu18172871)
Supplement: Supplementary file 1 [file nutrients-18-02871-s001.zip › nutrients-4478317-supplementary.pdf]

Supplementary Materials

Urban–Rural Disparities in School Lunch Food Composition

Figure S1. Three-tier urbanicity classification of Taiwan's 22 counties and cities. Metropolitan (dark grey, n = 7), suburban (mid grey, n = 6), and rural (light grey, n = 9) tiers follow the classification used throughout the analysis (see Table S2); red outlines mark the six counties of the February 2017 '3 Labels & 1 QR' pilot. Kinmen and Lienchiang (Matsu) counties are shown in insets.

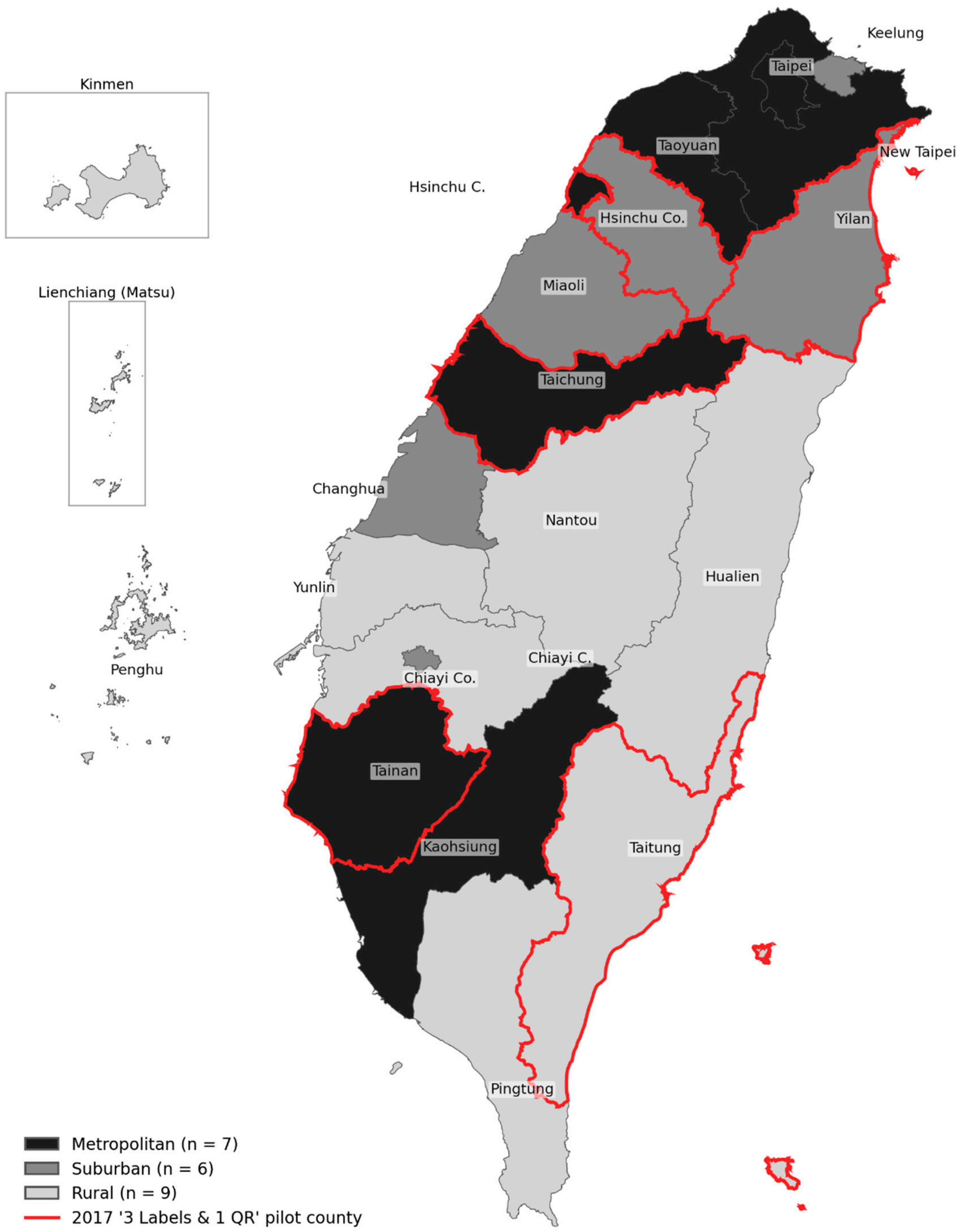

Text S1. The School Food Registry Platform

Taiwan's School Food Registry Platform (校園食材登錄平臺; https://fatraceschool.k12ea.gov.tw/), operated under the Ministry of Education's K-12 Education Administration, requires every public school lunch program—whether prepared by an on-site kitchen, a central kitchen, or a contracted caterer—to log each day's dishes and ingredient sources after lunch service. Logging is item-level: each menu line (dish name) constitutes one record, and ingredient entries carry certification fields for the '3 Labels & 1 QR' scheme. Complete daily logging is the administrative condition for claiming the central traceable-ingredient incentive described in Section 2.1, and the platform performs automated consistency checks against supplier records; compliance is therefore near-universal, and the Ministry of Education reports that certified traceable ingredients account for 98.05% of school-lunch ingredient items. The registry is publicly accessible through an open application programming interface, from which the 12,688,008 dish records analyzed in this study were retrieved (May 2024–April 2026; all 22 counties; 3,894 public schools). The platform thus functions simultaneously as a compliance instrument and as a census-level administrative data source on what Taiwanese schoolchildren are actually served.

Text S2. Policy Timeline of the '3 Labels & 1 QR' Program

• **2013 —** The Ministry of Education's School Lunch Food Content and Nutritional Standards are revised to restrict the supply of fish- and meat-based semi-processed items (e.g., fish/meat balls, sausages, ham, reconstituted nuggets).

• **February 2017 (second semester of SY 2016–17) —** The Council of Agriculture launches the '3 Labels & 1 QR' traceable-ingredient program in six pilot counties—Hsinchu County, Hsinchu City, Taichung City, Tainan City, Taitung County, and Yilan County—with a central incentive of NT$3.5 per student-meal (Executive Yuan, 2019).

• **September 2018 (SY 2018–19) —** Nationwide rollout to all 22 counties and cities (Executive Yuan, 2019).

• **December 2019 —** An Executive Yuan review reports an annual central budget of roughly NT$1.2 billion and a certified-label usage rate of 54% as of October 2019 (Executive Yuan, 2019).

• **28 December 2020 —** The Nutritional Standards are further revised: deep-fried items are capped at twice per week and sugary desserts at once per week, semi-processed products are to be minimized, and whole and unrefined staples are specified in the daily food-group composition (Ministry of Education, 2020).

• **2022 —** The Food and Agriculture Education Act is enacted; together with Article 23 of the School Health Act, it provides the statutory basis for the guideline on domestic traceable ingredients for school lunches. From May 2022, the incentive is raised to NT$10 per student per day (NT$14 for officially designated remote schools) (New Taipei City Government, 2022).

• **May 2024 – April 2026 —** Observation window of the present study; certified traceable ingredients account for 98.05% of school-lunch ingredient items (Ministry of Education press statement).

Sources are cited in full in the main-text reference list.

Table S1. Full four-step hierarchical regression results for all four dietary composition outcomes (main analysis, both school levels pooled; corrected pilot-county coding).

Entries are unstandardized coefficients in percentage points with HC3 heteroskedasticity-consistent standard errors in parentheses. All models are estimated by weighted least squares with the number of contributing schools per county-month as weights (N = 418 county-months in all models). The reference category for urbanicity is metropolitan counties; household disposable income, agricultural employment, and population density are standardized. Month fixed effects are included in all models; Step 1 contains month effects only and their coefficients are omitted for brevity. † $p < 0.10$, * $p < 0.05$, ** $p < 0.01$, *** $p < 0.001$.

Panel A. Plant-forward share

Predictor	Step 1	Step 2	Step 3	Step 4
Suburban (ref. metropolitan)	—	−0.553*** (0.138)	−0.445*** (0.132)	−0.496*** (0.145)
Rural (ref. metropolitan)	—	−0.868*** (0.114)	−0.506** (0.194)	−0.649*** (0.191)
Household disposable income (std.)	—	—	−0.345*** (0.075)	−0.325*** (0.070)
Agricultural employment (std.)	—	—	−0.410*** (0.074)	−0.412*** (0.069)
Population density (std.)	—	—	+0.062† (0.037)	−0.004 (0.050)
'3 Labels & 1 QR' pilot county	—	—	—	−0.412** (0.129)
R^2	0.011	0.166	0.231	0.261
ΔR^2	—	0.155***	0.065***	0.031***
ΔF (p)	—	36.98 (0.000)	11.08 (0.000)	16.28 (0.000)

Panel B. Organic share

Predictor	Step 1	Step 2	Step 3	Step 4
Suburban (ref. metropolitan)	—	−0.228 (0.485)	+1.314*** (0.398)	+1.552*** (0.363)
Rural (ref. metropolitan)	—	−0.535 (0.405)	+3.891*** (0.531)	+4.564*** (0.486)
Household disposable income (std.)	—	—	+2.963*** (0.216)	+2.868*** (0.189)
Agricultural employment (std.)	—	—	−0.012 (0.160)	−0.001 (0.140)
Population density (std.)	—	—	+0.080 (0.119)	+0.387* (0.151)
'3 Labels & 1 QR' pilot county	—	—	—	+1.931*** (0.374)
R^2	0.003	0.008	0.348	0.411
ΔR^2	—	0.005	0.341***	0.062***
ΔF (p)	—	1.04 (0.356)	68.66 (0.000)	41.65 (0.000)

Panel C. Processed share

Predictor	Step 1	Step 2	Step 3	Step 4
Suburban (ref. metropolitan)	—	−0.223*** (0.066)	−0.331*** (0.050)	−0.303*** (0.056)
Rural (ref. metropolitan)	—	+0.059 (0.075)	−0.314** (0.097)	−0.236* (0.112)
Household disposable income (std.)	—	—	−0.466*** (0.039)	−0.477*** (0.038)
Agricultural employment (std.)	—	—	−0.111* (0.046)	−0.109* (0.046)
Population density (std.)	—	—	+0.085*** (0.016)	+0.121*** (0.023)
'3 Labels & 1 QR' pilot county	—	—	—	+0.226** (0.082)
R^2	0.011	0.043	0.233	0.261
ΔR^2	—	0.032**	0.189***	0.029***
ΔF (p)	—	6.74 (0.001)	32.43 (0.000)	15.34 (0.000)

Panel D. Whole-grain share

Predictor	Step 1	Step 2	Step 3	Step 4
Suburban (ref. metropolitan)	—	−0.330† (0.197)	+0.350** (0.124)	+0.334* (0.133)
Rural (ref. metropolitan)	—	−0.189 (0.182)	+2.917*** (0.210)	+2.872*** (0.231)
Household disposable income (std.)	—	—	+1.210*** (0.076)	+1.216*** (0.079)

Predictor	Step 1	Step 2	Step 3	Step 4
Agricultural employment (std.)	—	—	−1.109*** (0.092)	−1.110*** (0.092)
Population density (std.)	—	—	−0.781*** (0.035)	−0.801*** (0.051)
'3 Labels & 1 QR' pilot county	—	—	—	−0.130 (0.150)
R^2	0.007	0.016	0.499	0.501
ΔR^2	—	0.009	0.483***	0.002
ΔF (p)	—	1.81 (0.165)	126.56 (0.000)	1.19 (0.276)

Table S2. County-level classification and structural covariates. Urbanicity tiers are those used throughout the analysis (metropolitan n = 7, suburban n = 6, rural n = 9). Mean household disposable income is from the 2024 Family Income and Expenditure Survey (NT$ thousand); values marked * (Kinmen and Lienchiang) are imputed from historical county data. ✓ marks the six counties of the February 2017 '3 Labels & 1 QR' pilot.

Tier	County/City	Income (NT$ 1,000)	2017 pilot
Metropolitan	Hsinchu City	1858.0	✓
Metropolitan	Taipei City	1803.2	
Metropolitan	Taoyuan City	1514.5	
Metropolitan	New Taipei City	1472.6	
Metropolitan	Taichung City	1427.4	✓
Metropolitan	Kaohsiung City	1379.7	
Metropolitan	Tainan City	1225.3	✓
Suburban	Hsinchu County	1868.8	✓
Suburban	Chiayi City	1456.0	
Suburban	Miaoli County	1392.8	
Suburban	Yilan County	1116.1	✓
Suburban	Changhua County	1100.0	
Suburban	Keelung City	1083.6	
Rural	Yunlin County	1104.0	
Rural	Pingtung County	1086.3	
Rural	Hualien County	1070.2	
Rural	Kinmen County	1050.0 *	
Rural	Nantou County	1048.9	
Rural	Chiayi County	983.3	
Rural	Lienchiang County	950.0 *	
Rural	Penghu County	927.8	
Rural	Taitung County	916.6	✓

Table S3. Hierarchical regressions re-estimated on elementary and junior-high schools only (compulsory-education tier; corrected pilot-county coding).

Every county-month cell retains at least one elementary/junior-high school meeting the five-serving-day threshold; the cell count (N = 418 in all models) is therefore unchanged, while cell composition and school-count weights reflect the restricted sample. Entries are unstandardized coefficients in percentage points with HC3 standard errors in parentheses; specifications match the main analysis. † $p < 0.10$, * $p < 0.05$, ** $p < 0.01$, *** $p < 0.001$. Focal urbanicity, income, and policy patterns replicate the pooled analysis; the rural coefficient for processed share does not reach significance in the restricted sample, though its direction and magnitude are consistent with the pooled estimate, and covariate coefficients vary somewhat across specifications, consistent with the sensitivity note in the main text.

Panel A. Plant-forward share

Predictor	Step 1	Step 2	Step 3	Step 4
Suburban (ref. metropolitan)	—	−0.657*** (0.145)	−0.502*** (0.142)	−0.543*** (0.153)
Rural (ref. metropolitan)	—	−0.991*** (0.125)	−0.509* (0.213)	−0.631** (0.207)
Household disposable income (std.)	—	—	−0.352*** (0.080)	−0.334*** (0.076)
Agricultural employment (std.)	—	—	−0.477*** (0.079)	−0.482*** (0.075)
Population density (std.)	—	—	+0.066† (0.039)	+0.006 (0.052)
'3 Labels & 1 QR' pilot county	—	—	—	−0.366** (0.136)
R^2	0.010	0.183	0.253	0.273
ΔR^2	—	0.173***	0.070***	0.020**
ΔF (p)	—	42.07 (0.000)	12.31 (0.000)	10.94 (0.001)

Panel B. Organic share

Predictor	Step 1	Step 2	Step 3	Step 4
Suburban (ref. metropolitan)	—	−0.256 (0.528)	+1.462*** (0.432)	+1.723*** (0.380)
Rural (ref. metropolitan)	—	−0.674 (0.439)	+4.117*** (0.589)	+4.885*** (0.513)
Household disposable income (std.)	—	—	+3.047*** (0.234)	+2.929*** (0.198)
Agricultural employment (std.)	—	—	−0.003 (0.177)	+0.029 (0.153)
Population density (std.)	—	—	+0.361** (0.125)	+0.737*** (0.156)
'3 Labels & 1 QR' pilot county	—	—	—	+2.310*** (0.391)
R^2	0.003	0.010	0.368	0.445
ΔR^2	—	0.007	0.358***	0.077***
ΔF (p)	—	1.44 (0.239)	74.40 (0.000)	54.40 (0.000)

Panel C. Processed share

Predictor	Step 1	Step 2	Step 3	Step 4
Suburban (ref. metropolitan)	—	−0.318*** (0.060)	−0.406*** (0.048)	−0.381*** (0.055)
Rural (ref. metropolitan)	—	+0.027 (0.077)	−0.266* (0.105)	−0.195 (0.122)
Household disposable income (std.)	—	—	−0.416*** (0.040)	−0.427*** (0.040)
Agricultural employment (std.)	—	—	−0.135** (0.047)	−0.132** (0.047)
Population density (std.)	—	—	+0.046** (0.017)	+0.081** (0.025)
'3 Labels & 1 QR' pilot county	—	—	—	+0.213* (0.087)
R^2	0.011	0.064	0.222	0.247
ΔR^2	—	0.053***	0.158***	0.025***
ΔF (p)	—	11.32 (0.000)	26.66 (0.000)	12.85 (0.000)

Panel D. Whole-grain share

Predictor	Step 1	Step 2	Step 3	Step 4
Suburban (ref. metropolitan)	—	−0.316 (0.202)	+0.410** (0.131)	+0.398** (0.138)

Predictor	Step 1	Step 2	Step 3	Step 4
Rural (ref. metropolitan)	—	−0.301 (0.186)	+2.920*** (0.233)	+2.886*** (0.250)
Household disposable income (std.)	—	—	+1.203*** (0.082)	+1.208*** (0.086)
Agricultural employment (std.)	—	—	−1.143*** (0.092)	−1.145*** (0.092)
Population density (std.)	—	—	−0.722*** (0.035)	−0.738*** (0.054)
'3 Labels & 1 QR' pilot county	—	—	—	−0.102 (0.167)
R^2	0.008	0.019	0.492	0.493
ΔR^2	—	0.012†	0.473***	0.001
ΔF (p)	—	2.34 (0.097)	122.15 (0.000)	0.69 (0.408)
